# Examination of Appendiceal Neoplasms—A Retrospective, Single-Centre, Cohort Study

**DOI:** 10.3390/cancers17244028

**Published:** 2025-12-18

**Authors:** Berkenye Csonka, Tamás Lantos, Anita Sejben

**Affiliations:** 1 Department of Pathology, University of Szeged, 6725 Szeged, Hungary; 2 Department of Medical Physics and Informatics, University of Szeged, 6720 Szeged, Hungary

**Keywords:** appendix, appendiceal tumours, appendiceal neoplasms

## Abstract

Background: Appendiceal neoplasms are rare, but recent data indicate a rising trend, particularly in patients under the age of 50. These tumours are often diagnosed incidentally during histopathological examination. This study has aimed to examine the incidence and histological subtypes of appendiceal neoplasms in a Southern Hungarian population. Methods: Our study included neoplastic appendix specimens processed at the University of Szeged between 2014 and 2023. Results: Neoplasms were identified in 71 cases from 3640 appendectomies (1.9%). Benign lesions were present in 37% of cases (*n* = 26), with the most common subtype being the sessile serrated lesion (*n* = 20). Mucinous and malignant neoplasms were found in 63% of cases (*n* = 45), most frequently low-grade appendiceal mucinous neoplasm (*n* = 19), followed by neuroendocrine tumour (*n* = 17). Notably, colorectal neoplasm was identified in 50% of benign, and 42.2% of mucinous and malignant cases during a mean follow-up of 33.4 months. Significant associations were found between histological subtype and age (*p* = 0.022), complete resection (*p* = 0.012), presence of vascular invasion (*p* = 0.007), and localisation of potentially associated colorectal carcinoma (*p* = 0.018). Additionally, tumour dignity showed significant correlations with tumour, node, metastasis (TNM) stage (*p* < 0.001), vascular invasion (*p* = 0.017), and lastly, occurrence (*p* = 0.031) and localisation (*p* = 0.003) of associated colorectal carcinoma. Conclusions: The prevalence and characteristics observed in this Southern Hungarian population were consistent with international data, although raw case numbers suggested an upward trend. The high rate of associated colorectal neoplasms underscores the importance of thorough pathological evaluation and long-term surveillance.

## Simple Summary

Appendiceal neoplasms are rare but showing increasing tendency in incidence, particularly in individuals under 50. In a study of 3640 appendectomy specimens from the University of Szeged (2014–2023), neoplasms were found in 1.9% of cases. Of these, 37% were benign—most commonly sessile serrated lesions—and 63% were mucinous or malignant, with low-grade appendiceal mucinous neoplasms and neuroendocrine tumours being most frequent. Patients with benign tumours were generally older, and nearly half of all neoplasm cases were associated with colorectal carcinoma. Significant correlations were observed between tumour characteristics and clinical outcomes. The findings highlight the need for heightened awareness and follow-up in managing appendiceal neoplasms.

## 1. Introduction

Primary and secondary neoplasms of the appendix are exceedingly rare, with a prevalence of 0.1–1.6 cases per 100,000 individuals. Among gastrointestinal tract neoplasms, they account for less than 1% of cases and are typically incidental findings, most often discovered during histopathological evaluation following appendectomy. Neoplasms are found in approximately 1% of all appendectomy specimens [1,2]. Their prevalence may vary by region and histological subtype, with appendiceal neuroendocrine tumours (NETs) being the most common [3]. Retrospective studies have demonstrated an increasing incidence of appendiceal neoplasms, affecting all histological subtypes and age groups. NETs have shown a particularly steep rise in incidence compared to other subtypes, especially among younger patients (<50 years). A study conducted in the United States and Canada found that the incidence of certain histological types has nearly tripled over the past 40 years [4]. This rising trend has also been confirmed by a similar analysis in the United Kingdom. The reasons for this increase are not yet fully understood and are likely multifactorial. Potential contributing factors include changes in healthcare practices—such as more frequent appendectomies in young patients, routine histological processing, and an increased number of tissue sections—as well as patient-related factors such as lifestyle changes and the rising prevalence of obesity [5].

The aetiology of appendiceal neoplasms remains mostly unknown. Identified risk factors include tobacco use, advanced age, female sex, and certain health conditions such as pernicious anaemia or atrophic gastritis [6]. It has been hypothesised that postinflammatory changes can induce the formation of hyperplastic polyps (HPs) [1].

Considering all subtypes of appendiceal neoplasms, the average age at diagnosis is between 50 and 55 years. Appendiceal adenocarcinomas tend to occur in older patients (average age 62–65), whereas neuroendocrine neoplasms (NENs) are more commonly diagnosed in younger individuals, peaking around the age of 40. Females are slightly overrepresented among patients, although this trend is reversed among cases of appendiceal adenocarcinoma, which are more common in males [7,8,9].

The clinical presentation is often nonspecific and may not suggest the underlying disease clearly. Appendiceal neoplasms are frequently asymptomatic and are typically discovered only at advanced stages. They may mimic acute appendicitis, presenting with right lower quadrant abdominal pain or a palpable mass in the case of a large tumour. In women, symptoms may resemble gynaecological disorders such as ovarian cyst torsion or rupture, or endometriosis. They can also cause obstructive symptoms or, less commonly, gastrointestinal bleeding. In metastatic cases, symptoms may manifest in the affected organs [9,10,11]. A single report described bowel obstruction as a rare presentation of an appendiceal mucinous neoplasm in an elderly patient [12]. Due to these nonspecific signs, preoperative diagnosis of appendiceal neoplasms is often difficult.

If symptomatic, patients are further evaluated using radiological methods, often leading to surgical intervention depending on the imaging findings. Radiological features may include localised soft tissue or fluid accumulation around the appendix, and sometimes calcifications in the appendiceal wall [9]. In cases involving peritoneal dissemination, ascites may also be present. The most common imaging modality is ultrasound, while computed tomography (CT) is used less frequently [2]. Occasionally, follow-up includes endoscopic examination during which, rarely, but pathognomically, a mucinous leak through the orifice of the appendix may be seen in cases of mucin-producing neoplasms of the appendix [13]. This is consistent with data showing that surgical suspicion of neoplasm is also raised in only one in seven confirmed cases—similar to CT [14].

Generally, appendiceal neoplasms follow an indolent course. A collective prognosis for appendiceal neoplasms cannot be defined with certainty; the long-term survival has been reported between 10 and 90% [1]. However, aggressive phenotypes can directly invade the peritoneum, causing peritoneal carcinomatosis, or rupture and disseminate within the abdominal cavity. Non-mucinous appendiceal adenocarcinomas typically metastasise to lymph nodes, the liver, lungs, and peritoneum [7]. The least favourable overall survival (OS) among primary appendiceal tumours has been reported for signet-ring cell adenocarcinomas, with a 5-year survival of 63.2%. Poor prognosis has been associated with peritoneal invasion and the presence of neoplastic cells in ascites [15].

The current, 5th edition of the World Health Organization (WHO) Classification of Tumours of the Digestive System, published in 2019, distinguishes five types of appendiceal neoplasms, including serrated lesions and polyps, appendiceal mucinous neoplasms (AMNs), adenocarcinoma, goblet cell adenocarcinoma, and NENs [9]. Ultimately, the definitive diagnosis is established by the pathologist. Even at this stage, differential diagnosis can be challenging. Inflammatory processes can mimic neoplastic lesions on a microscopic level as well; for example, diverticulitis may resemble low-grade appendiceal mucinous neoplasms (LAMN), and xanthogranulomatous inflammation may mimic malignancy [16]. Furthermore, it is not always clear whether a neoplastic lesion found in the appendix is primary or a metastasis from another site. This distinction is especially important in the case of mucinous ovarian neoplasms, which can mimic AMNs but require different treatment strategies [17].

To date, no study has examined the incidence and histological subtypes of appendiceal neoplasms in the Hungarian population.

## 2. Materials and Methods

A database was created of cases coded with International Classification of Diseases, Tenth Revision (ICD-10) codes D1210 (Benign neoplasm of the appendix), D3730 (Neoplasm of uncertain or unknown behaviour of the appendix), and C1810 (Malignant neoplasm of the appendix) between 2014 and 2023. Our consecutive case series included only patients with primary appendiceal neoplasms, and whose specimens were processed at the Department of Pathology, University of Szeged, and were all re-reviewed by a fellowship-trained gastrointestinal pathologist (AS).

For each case, the following clinicopathological parameters were recorded: patient’s gender, their age at the time of diagnosis and at the end of follow-up, histological tumour subtype, largest macroscopic diameter, tumour grade, complete or incomplete resection, and, in malignant cases, tumour stage and presence of any specific patterns of spread. Additionally, we documented whether the patient developed a benign or malignant colorectal neoplasm (CRN) after the diagnosis of the appendiceal tumour. For malignant CRNs, tumour localisation has also been recorded.

OS was defined as the time from the diagnosis of the appendiceal tumour to the last hospital visit, death, or the end of follow-up.

For statistical analyses, we used Fisher’s exact test, log-rank test, Mann–Whitney U test, and linear regression. Kaplan–Meier analysis was performed to evaluate survival data. A result was considered statistically significant if *p* < 0.05.

Our study was approved by the Medical Research Council (ETT TUKEB) (BM/28834-1/2024), as well as by the Regional and Institutional Research Ethics Committee for Human Medical Biology, University of Szeged (49/2025-SZTE-IKEB).

## 3. Results

### 3.1. General Cohort Data

Between 2014 and 2023, a total of 3640 appendectomies were performed at the University of Szeged, with all specimens processed at the Department of Pathology, University of Szeged. From these, 71 neoplastic cases were identified and included in our database, comprising 26 benign (37%) and 45 either mucinous or malignant (63%) neoplasms. A slight female predominance was observed among the neoplastic cases (*n* = 40; 56.34%). Clinically, all patients presented with appendicitis.

The mean follow-up period was 33.4 months (median: 30 months; range: 0–108 months).

**Figure 1:**
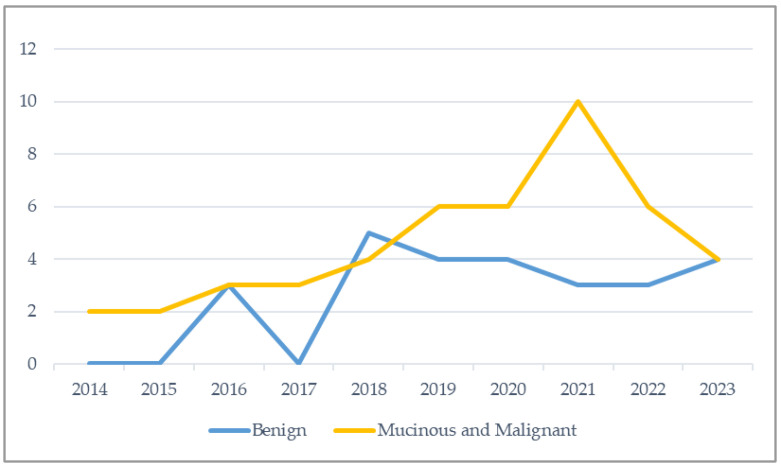
Annual case numbers of benign vs. mucinous and malignant appendiceal tumours during the course of the study.

**Figure 2:**
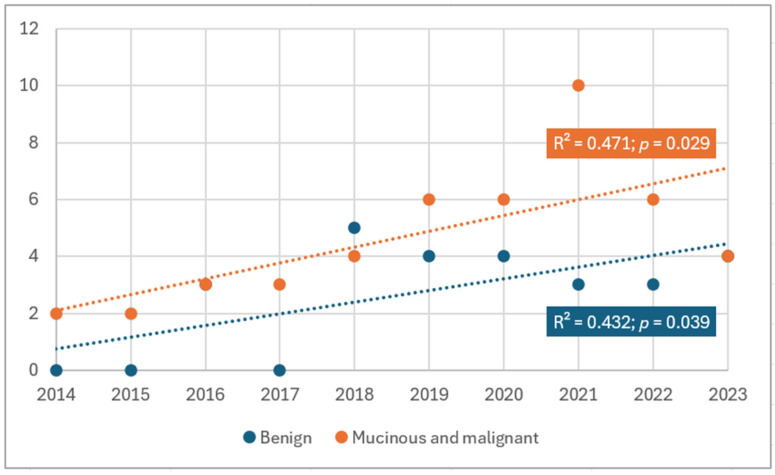
Annual trends in the number of benign vs. mucinous and malignant tumours. Fitted dashed lines were obtained from linear regression. Model fit (R-squared) and the corresponding *p*-values are displayed.

The annual distribution of benign and mucinous or malignant tumours during the study interval, based on the year of diagnosis, is shown in Figure 1. The data indicate a modest increase in the number of cases over time, with a more pronounced rise in mucinous and malignant diagnoses compared to benign ones. Linear trend analysis fitted to the number of tumours reflected statistically significant increase during the study period (Figure 2).

**Figure 3:**
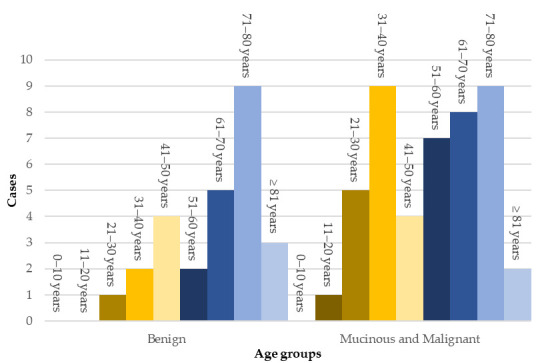
Age distribution of benign vs. mucinous and malignant appendiceal tumours.

The age distribution of patients diagnosed with benign vs. mucinous or malignant appendiceal neoplasms was examined, as well, and presented by decades in Figure 3.

A pronounced peak in the incidence of benign tumours was observed in the 71–80 years age group, whereas the distribution of mucinous and malignant tumours appeared more variable across different age groups.

**Figure 4:**
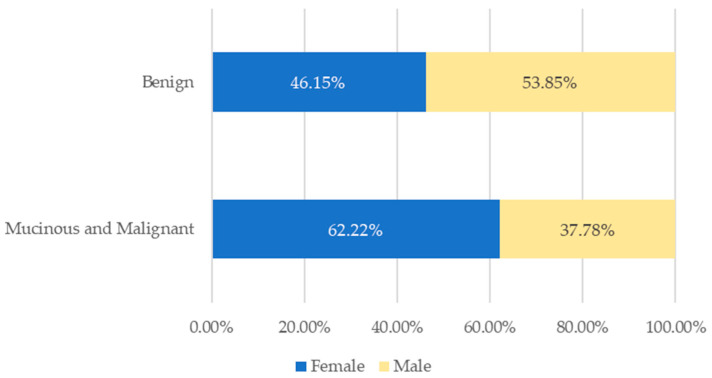
Gender distribution of benign vs. mucinous and malignant appendiceal tumours.

Regarding the major histopathological subgroups among the cases—benign neoplasms vs. the four main groups, including AMN, NEN, adenocarcinoma, and signet-ring cell adenocarcinoma, the following gender distributions were observed. Among benign neoplasms, 46.15% (*n* = 12) of cases were female, while in mucinous and malignant neoplasms, females comprised the majority, 62.22% (*n* = 28) of cases. Within the mucinous and malignant group, 70% (*n* = 14) of AMN cases were female, 61.10% (*n* = 11) of NEN cases were female, and 100% (*n* = 3) of signet-ring cell adenocarcinoma cases were female. On the other hand, all adenocarcinoma patients were male (100%; *n* = 4). The gender distribution of benign vs. mucinous and malignant tumours is illustrated in Figure 4.

### 3.2. Examination of Benign Appendiceal Neoplasms

A total of 26 cases (36.62%) were diagnosed as benign neoplasms. This corresponds to a prevalence of 7.14 per 1000 appendectomies. The male-to-female ratio was 14:12, indicating no clear gender predominance. The mean age of patients at diagnosis was 63.9 years (median: 69.5; range: 30–90 years).

**Figure 5:**
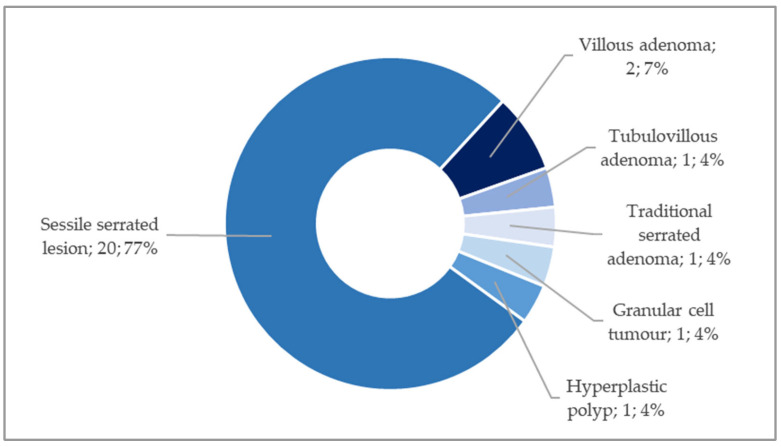
Distribution of benign histological subtypes.

Regarding histological subtypes, sessile serrated lesions (SSLs) were predominantly observed (*n* = 20; 76.93%). Additionally, conventional adenomas were diagnosed in three cases (11.54%), of which two were villous and one was a tubulovillous adenoma (TVA), all exhibiting low-grade dysplasia. One case presented with a traditional serrated adenoma (TSA) (3.85%), another with a HP (3.85%), and a case was diagnosed as a granular cell tumour (3.85%). The distribution of histological subtypes is graphically illustrated in Figure 5.

The resection was complete (R0) in all cases (*n* = 26). The mean OS from the time of diagnosis was 25.2 months (median: 14 months; range: 0–101 months). Altogether, 13 patients (50%) were lost to follow-up. Within the follow-up period, three patients died. The average survival time until the end of follow-up was 24 months (median: 24 months; range: 5–43 months).

**Figure 6:**
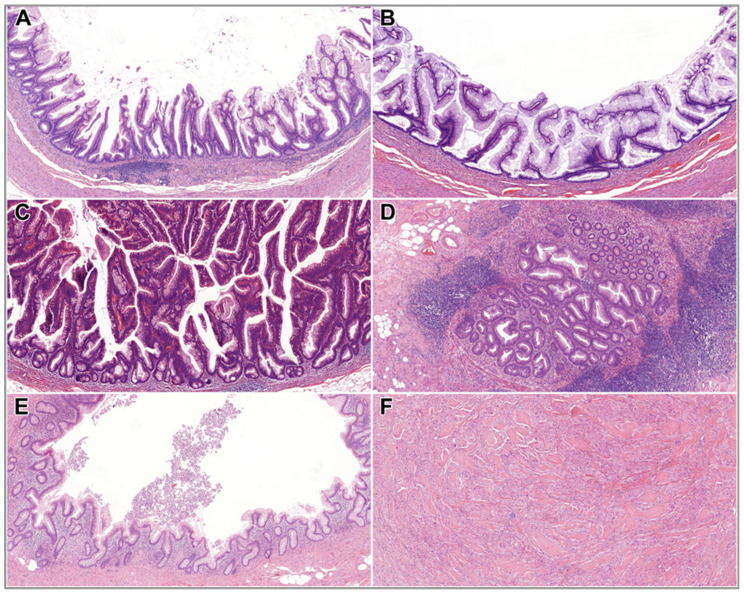
Histological features of benign appendiceal neoplasms. (**A**) SSL (HE, 5x); (**B**) Villous adenoma, hypermucinous variant (HE, 5x); (**C**) TVA (HE, 5x); (**D**) TSA (HE, 5x); (**E**) HP (HE, 5x); (**F**) Granular cell tumour (HE, 5x). Abbreviations: HE—hematoxylin and eosin; HP—hyperplastic polyp; SSL—sessile serrated lesion; TSA—traditional serrated adenoma; TVA—tubulovillous adenoma.

The co-occurrence of CRN among benign cases was as follows: 50% of the cases (*n* = 13) presented with either benign or malignant CRN. Among the CRNs, 92.31% (*n* = 12) were malignant, while 7.69% (*n* = 1) were benign. In two cases, multiple colorectal adenocarcinomas were present, affecting both the right and left colon. The right colon was more frequently involved, accounting for 64.29% (*n* = 9) of the colorectal carcinomas (CRCs), while 35.71% (*n* = 5) involved the left colon. The mean age of patients diagnosed with both benign appendiceal neoplasm and CRC was 74.8 years (median: 73.5; range: 69–85 years). Summary data for benign cases are provided in Table 1 and Table 2, while their histological morphology is illustrated in Figure 6.

### 3.3. Examination of Mucinous and Malignant Appendiceal Neoplasms

**Figure 7:**
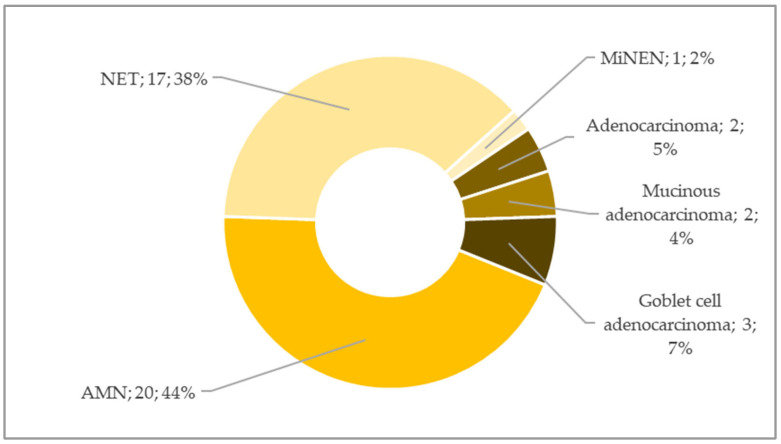
Distribution of mucinous and malignant neoplasms’ histological subtypes. Abbreviations: AMN—appendiceal mucinous neoplasm; MiNEN—mixed neuroendocrine-non-neuroendocrine neoplasm; NET—neuroendocrine tumour.

Mucinous and malignant neoplasms were identified in 45 cases (63.4%), corresponding to a prevalence of 12.36 per 1000 appendectomies. The male-to-female ratio was 17:28, indicating a clear female predominance. The mean age of patients at diagnosis was 53.6 years (median: 55 years; range: 16–88 years). Histologically, 20 cases (44.4%) were classified as AMN, of which 11 (55%) were in situ. Additionally, one case (5%) was staged as pT1, one case (5%) as pT3, and seven cases (35%) as pT4. Tumour resection was complete (R0) in 19 cases (95%), while 1 case (5%) was incompletely resected. Regarding grade, the vast majority of AMNs (95%, *n* = 19) were LAMN, with one case diagnosed as high-grade appendiceal mucinous neoplasm (HAMN). Altogether 18 cases were classified as NENs, among which NETs were diagnosed in 17 cases (37.8%) and 1 case (2.22%) was identified as mixed neuroendocrine-non-neuroendocrine neoplasm (MiNEN). Adenocarcinomas were diagnosed in four cases (8.9%), among which two were mucinous. Finally, three cases (6.7%) were identified as signet-ring cell adenocarcinomas. The distribution of histological subtypes within the mucinous and malignant group is illustrated in Figure 7.

**Figure 8:**
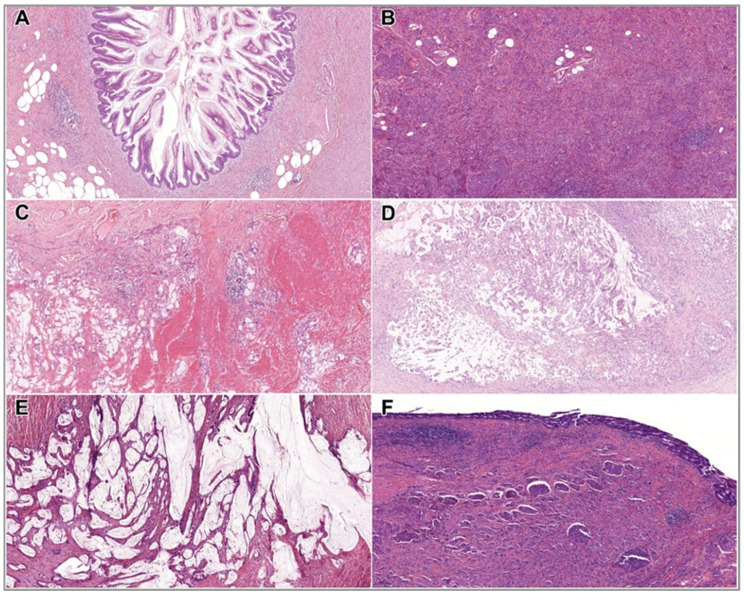
Histological features of mucinous and malignant appendiceal neoplasms. (**A**) HAMN (HE, 5x); (**B**) NET (HE, 5x); (**C**) goblet cell adenocarcinoma (HE, 5x); (**D**) adenocarcinoma (HE, 5x); (**E**) mucinous adenocarcinoma (HE, 5x); (**F**) MiNEN (HE, 5x). Abbreviations: HAMN—high-grade appendiceal mucinous neoplasm; HE—hematoxylin and eosin; MiNEN—mixed neuroendocrine-non-neuroendocrine neoplasm; NET—neuroendocrine tumour.

Complete (R0) resection was achieved in 91.1% of mucinous and malignant neoplasms (*n* = 41), while 8.9% (*n* = 4) underwent R1 resection. The mean OS was 38.1 months (median: 34 months; range: 0–108 months). In total, 24.44% of patients (11 cases) were lost to follow-up. As of 31 August 2024, three patients had died, with deaths occurring 1 (*n* = 2) and 82 months after diagnosis, respectively. The clinicopathological data of malignant cases are summarised in Table 3. Histological morphology of the malignant cases is illustrated in Figure 8.

Regarding the investigation of CRNs occurring among mucinous and malignant neoplasms, the following observations were made. CRNs were present in 42.2% of cases (*n* = 19). Of these CRNs, 57.9% (*n* = 11) were malignant, while 42.1% (*n* = 8) were benign. Among CRCs, 18.2% (*n* = 2) had unknown localisation, 54.55% (*n* = 6) involved the right colon, and 27.27% (*n* = 3) involved the left colon. The mean age of patients diagnosed with both malignant appendiceal neoplasm and CRC was 64.4 years (median: 65 years; range: 52–79 years). A summary of these data is presented in Table 4. Comparative clinicopathological characteristics of the benign vs. mucinous and malignant subgroups are summarised in Table 5.

### 3.4. Results of Statistical Analysis

A significant association was observed between histological subtype and age (*p* = 0.022), complete resection (*p* = 0.012), presence of venous invasion (*p* = 0.007), as well as the localisation of potentially associated CRC (*p* = 0.018). Furthermore, tumour behaviour was significantly associated with tumour, node, metastasis stage (TNM) (*p* < 0.001), presence of venous invasion (*p* = 0.017), development of associated CRC (*p* = 0.031), and its localisation (*p* = 0.003).

No significant correlation was found between histological subtype of appendiceal tumours and gender (*p* = 0.486), largest tumour diameter (*p* = 0.155), occurrence of potentially associated CRC (*p* = 0.082), or OS (*p* = 0.236). Similarly, tumour dignity showed no significant association with gender (*p* = 0.27), age (*p* = 0.1), largest tumour diameter (*p* = 0.292), grade (*p* = 0.76), complete resection (*p* = 0.101), or OS (*p* = 0.401).

**Figure 9:**
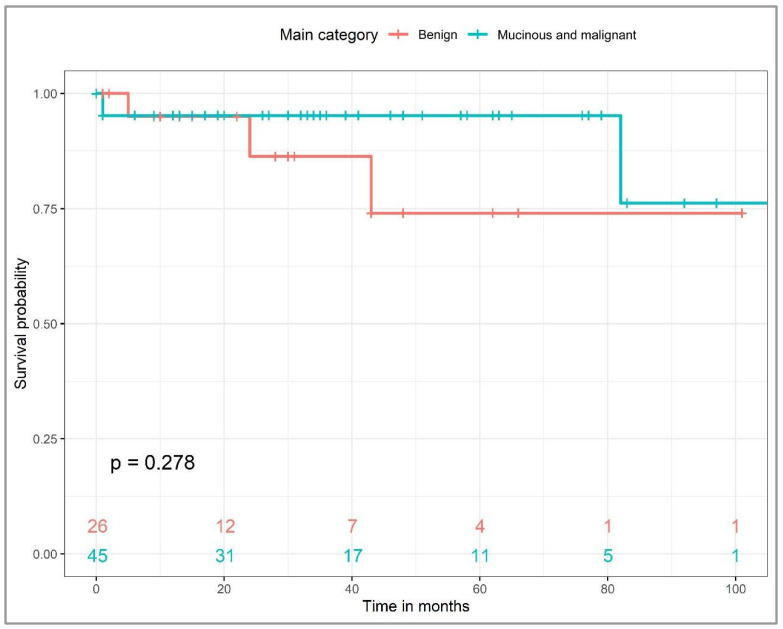
Kaplan–Meier analysis of OS among benign and mucinous or malignant tumours. Abbreviations: OS—overall survival.

Distinct survival times were observed across histological subtypes, with median survival of 22 months for cases diagnosed with SSLs, 31 months for AMNs, and 54.5 months for NENs. Kaplan–Meier analysis showed no significant difference in OS between benign and mucinous or malignant tumours. The Kaplan–Meier survival curves are illustrated in Figure 9.

## 4. Discussion

Appendix neoplasms form a rare and diverse group of diseases, with a combined prevalence of 0.1–1.6 cases per 100,000 individuals [2]. The classification of appendix neoplasms has undergone numerous changes over recent decades; currently, the WHO 2019 classification is authoritative in pathological diagnostics. The WHO identified five major groups: serrated lesions and polyps belonging to benign neoplasms, mucinous neoplasms, and malignant neoplasms, including adenocarcinoma, goblet cell adenocarcinoma, and NENs [9]. Due to the many previous classifications and diverse nomenclature, comparability of the data presented in the literature using different terminology is limited.

The most common histological type is NET, and generally, malignant neoplasms are more frequent than benign ones. This may be because benign lesions are less frequently registered [3,18,19,20]. Worldwide, an increase in the incidence of appendix neoplasms has been reported, including a widening age range, with a rising proportion of younger patients, whereas previously, appendix neoplasms mainly affected those over 50 years of age [4,5].

Clinically, appendix neoplasms are most often asymptomatic; they can cause appendicitis-like symptoms, but in most cases, they are incidentally found during histological examination [10,11]. Diagnostic difficulty arises due to their asymptomatic nature, small size, and rare detectability on ultrasound [21]. Bahmad et al. raised the question of whether diagnosing incidental appendix neoplasms has clinical significance, since most early-stage and curative treatments are already accomplished by the appendectomy itself at diagnosis [21].

In our study, we evaluated the occurrence and clinicopathological features of appendix neoplasms based on appendectomy specimens processed at the Department of Pathology, University of Szeged, between 2014 and 2023. During the study period, 3640 appendectomy specimens were analysed, of which neoplasms were identified in 71 cases. This corresponds to 19.5 neoplasms per 1000 appendectomies. This roughly matches the international literature reporting a prevalence of 7.9–21.38‰ [2,22,23]. An overall increase in case numbers was observed over the study period, with the rise being more pronounced for mucinous and malignant diagnoses than for benign conditions. It remains unclear whether this represents a true epidemiological shift. Because all surgical specimens have been fully embedded during macroscopic examination throughout the study period, the observed increase is unlikely to be attributable to changes in detection practices. One outlier data point is Bahmad’s American study, which reported a prevalence of 51.66‰ between 2010 and 2020. The author suggests this might be due to very accurate pathological analysis, although methodological details about grossing in other sources are lacking, making comparison difficult [21]. The raw case numbers over the years in our data show an increasing trend, consistent with international tendencies. It can also be said that the number of patients under 50 years old shows some increase.

The collective prevalence of benign neoplasms was 7.14‰, which is several times higher than the 1.5‰ incidence reported by Connor et al. in 1998 [18]. The most common benign neoplasm was SSL, with a prevalence of 5.22‰. Köhler’s study reported a lower prevalence of 1.14‰, while Núñez-Rocha’s study described a similar prevalence of 4.01‰ [1,23]. The average age of benign cases at diagnosis was significantly higher than in mucinous and malignant cases, at 69.5 years; this falls within the 5th–7th decade interval published in earlier studies [14,22]. Several studies have reported a female predominance in benign cases; however, our study, based on 26 cases, did not confirm this [20,23]. The 52.6% presence of CRN simultaneously occurring with SSLs corresponds to the frequent co-occurrence noted by Carr [16]. It can also be stated that CRN occurred significantly more often alongside benign neoplasms. Given that SSLs constitute the majority of benign appendiceal lesions in our cohort, and that most synchronous CRNs are right-sided, the results could be interpreted within the context of the serrated neoplastic pathway; however, it remains unclear. Within our cohort, a single patient was subsequently diagnosed with an SSL located outside the caecum, and another with a serrated adenocarcinoma, also arising outside the caecum. All remaining cases represented conventional adenomas or colorectal adenocarcinomas. It should be noted that both benign and malignant CRNs occur frequently in the general Hungarian population; therefore, the co-occurrence may only be due to older age. Regarding tumour sizes, benign neoplasms were generally under 10 mm, with one TVA exceeding 20 mm. Survival of cases with benign neoplasms during the study period was worse than that of malignant neoplasms, which may be explained by the older age of the benign patient group and significantly greater loss to follow-up, distorting survival data. It is important to highlight that these patients may have been registered solely because of the surgery, with no further appearances, as no adjuvant therapy was necessary.

The combined prevalence of mucinous and malignant neoplasms was 12.36‰, which is nearly identical to the 13.7‰ found in a Turkish study covering 2013–2018 [24]. The increasing prevalence of mucinous and malignant neoplasms is suggested by an earlier 1998 study that found only 2.51‰ incidence [18]. In our population, the most frequent mucinous or malignant neoplasms were AMNs, with LAMN constituting 42.22%; the second most common type was NET at 37.78%.

In the study of Köhler et al., LAMN was also the most frequent appendix neoplasm, whereas in four other studies, NET was the most common neoplasm [1,3,8,24,25]. These small differences are likely better evaluated in larger samples. Our results on gender distribution match international data. Overall, a slight female predominance was observed among malignant cases (62.22%); within this, AMN and NEN cases showed female predominance, whereas adenocarcinomas showed male predominance based on low case numbers. The largest diameters of AMNs were significantly larger than those of adenocarcinomas, consistent with CT findings described by Leonards, who noted mucin-producing neoplasms tend to be larger on average than non-mucinous ones [20]. The average age at diagnosis of NEN cases was 42.5 years, consistent with the reported age-range of 38–48 years, and significantly younger than other histological types [10].

In our study, CRN was present in 42% of mucinous and malignant cases—in 75% of adenocarcinomas and 44.4% of NENs. That is much higher than the 10% reported by Connor in 1998 and the 22% reported by Rossi based on 1997–2003 data [18,19]. This may relate to lifestyle factors in the Hungarian population, but could also reflect a broader increase in concurrent appendix and CRNs beyond just the Hungarian population. In any case, this supports the recommendation to perform a colonoscopy after diagnosis of an appendix neoplasm to search for simultaneous CRN [16,20].

Significant correlations were observed between histological subtype and age (*p* = 0.022), complete resection (*p* = 0.012), presence of venous invasion (*p* = 0.007), and potential associated CRC localisation (*p* = 0.018). Additionally, tumour grade correlated with TNM stage (*p* < 0.001), presence of venous invasion (*p* = 0.017), associated CRC development (*p* = 0.031), and localisation (*p* = 0.003).

Regarding age, a significant difference was noted between NETs (median: 48.5) and serrated lesions (median: 72). There is limited literature on the clinicopathology of benign appendix neoplasms. Núñez-Rocha et al. reported a mean age of 46 based on 12 cases, Chezar and Minoo reported 59.7 years based on 20 cases for benign appendix neoplasms, which differ considerably from our median age findings [23,26]. Our NET results align with international data, reporting a mean age of 35.5–51.1 years [11,24,26]. Complete resection was observed for benign tumours and NETs, but not for LAMN/HAMN or adenocarcinoma. Retrospective clinicopathological reports show no clear difference in histological type and complete resection, with similar 90.9–97% R0 resection rates among subtypes, though resection results were not directly compared [24,25,27]. Rencuzogullari et al. found no significant difference; however, Sahin et al. reported only one-third of their adenocarcinoma cases with complete resections (*n* = 2/6) [28,29]. Venous invasion was observed only in mucinous and malignant tumours. Hara et al. reported no histological evidence of venous invasion in LAMN and adenocarcinoma cases [30]. Associated CRC was mainly found in the right colon. TNM staging applies only to mucinous and malignant tumours, explaining the statistical correlations with the presence of venous invasion. Potentially associated CRC was found in nearly half of benign cases, and about one-third of mucinous and malignant appendix tumours; in both groups, CRC was mainly located in the right colon. We found no clinicopathological literature data about concurrent appendix and CRNs and their localisation.

Our study was carried out with the following limitations. Our review was restricted to appendectomy specimens for which a preliminary benign or malignant diagnosis had been assigned. Cases identified as having inaccurate diagnostic coding or misclassification were subsequently excluded from the dataset. Case numbers were unevenly distributed among histological groups, making valid statistical conclusions impossible for groups of 1–3 cases; these require larger population studies. Due to the fact that solely univariable comparisons have been carried out, given the relatively small sample size, analyses are exploratory, univariable, and not adjusted for multiple testing. Significant associations should be interpreted cautiously due to multiple comparisons and limited power. A major limitation regarding survival time was the significant loss of patients to follow-up—50% in benign cases and 24% in mucinous and malignant cases—which likely resulted in distortion of survival data. The single-centre nature of the cohort might be a limitation, as well, and might be a possible referral bias, as well as the absence of molecular data, which are increasingly relevant for appendiceal neoplasm classification.

Strengths of our research is that, to our knowledge, this is the first single-centre report from Hungary, providing baseline data for future studies, and the consecutive nature, allowing examination of several rare tumour types.

This clinicopathological study showed that benign neoplasms are characteristic of older populations, but the number of neoplasms diagnosed under age 50 is also increasing. Even though this study solely represents data of the Southern Hungarian population, the relatively high rate of concurrent CRN might indicate the further need to perform routine colonoscopy after appendix neoplasm diagnosis. In the examined population, SSL, AMN, and NET were present in high numbers. The significantly larger size of AMN cases calls for increased surgical caution, as rupture of these neoplasms carries the risk of pseudomyxoma peritonei development. NET cases typically affect younger patients, so the possibility of neoplasm should not be dismissed in patients under 40 years old. Overall, large population studies are needed to reliably identify survival, complications, and risk factors of appendix neoplasms.

## 5. Conclusions

This clinicopathological study found that while benign appendiceal neoplasms are more common in older individuals, cases in patients under 50 are rising. A notably high rate of concurrent CRN in the Hungarian cohort supports recommending routine colonoscopy following diagnosis. SSLs, AMNs, and NETs were the most common subtypes. Due to the larger size of AMNs and their risk of causing pseudomyxoma peritonei if ruptured, careful surgical management is advised. NETs, often found in younger patients, highlight the need to consider the possibility of a neoplasm even in those aged under 40 years. Larger studies are needed to clarify survival outcomes, complications, and risk factors.

## Figures and Tables

**Table 1:** Clinicopathological data of benign appendiceal neoplasms.

Histological Subtype	Number of Cases	Mean Age (Year) ± SD	Male/Female Ratio	Complete Resection	Mean Survival (Month) ± SD	Prevalence *
SSL	20 (76.92%)	63.15 ± 16.95	11:9	R0	30.15 ± 27.47	5.49‰
Villous adenoma	2 (7.69%)	75 ± 1.4	1:1	R0	19.5 ± 6.4	0.55‰
TVA	1 (3.85%)	72	0:1	R0	0	0.27‰
TSA	1 (3.85%)	84	0:1	R0	13	0.27‰
HP	1 (3.85%)	49	1:0	R0	0	0.27‰
Granular cell tumour	1 (3.85%)	44	1:0	R0	0	0.27‰

Abbreviations: HP—hyperplastic polyp; SD—standard deviation; SSL—sessile serrated lesion; TSA—traditional serrated adenoma; TVA—tubulovillous adenoma. * Prevalence calculated for appendectomies.

**Table 2:** Presence of CRN among cases diagnosed with benign appendiceal neoplasm.

Association of CRN with Benign Appendiceal Neoplasms				
Histological Subtype	Number of Cases	Presence of CRN	Benign	Malignant
SSL	20	55% (*n* = 11)	9% (*n* = 1)	91% (*n* = 10)
Villous adenoma	2	50% (*n* = 1)	0% (*n* = 0)	100% (*n* = 1)
TVA	1	0% (*n* = 0)	-	-
TSA	1	100% (*n* = 1)	0% (*n* = 1)	100% (*n* = 1)
HP	1	0% (*n* = 0)	-	-
Granular cell tumour	1	0% (*n* = 0)	-	-

Abbreviations: CRN—colorectal neoplasm; HP—hyperplastic polyp; SSL—sessile serrated lesion; TSA—traditional serrated adenoma; TVA—tubulovillous adenoma.

**Table 3:** Clinicopathological data of mucinous and malignant appendiceal neoplasms.

Histological Type	Number of Cases	pT	M	Spread	Mean Age (Year) ± SD	Male/ Female Ratio	Complete Resection	Mean Survival (Month) ± SD	Prevalence *
AMN	20 (44.44%)	In situ: 55% (*n* = 11) T1: 5% (*n* = 1) T3: 5% (*n* = 1) T4: 35% (*n* = 7)	M1: 10% (*n* = 2)	*-*	57.2 ± 15.4	5:15	R0: 95% (*n* = 19) R1: 5% (*n* = 1)	31.5 ± 25.5	5.49‰
NET	17 (37.78%)	T1: 64.7% (*n* = 11) T2: 5.9% (*n* = 1) T3: 17.7% (*n* = 3) T4: 11.8% (*n* = 2)	-	1 vascular	46 ± 20.4	7:10	R0	53.5 ± 30	4.67‰
Goblet cell adenocarcinoma	3 (6.67%)	T3: 66.7% (*n* = 2) T4: 33.3% (*n* = 1)	M1: 33.3% (*n* = 1)	2 lymphatic 1 perineural	58 ± 22.5	0:3	R0: 33.3% (*n* = 1) R1: 66.7% (*n* = 2)	24.7 ± 9	0.82‰
Adenocarcinoma	2 (4.44%)	NA **: 50% (*n* = 1) T4: 50% (*n* = 1)	-	1 vascular 1 lymphatic	64 ± 17	2:0	R0: 50% (*n* = 1) R1: 50% (*n* = 1)	0.5 ± 0.7	0.55‰
Mucinous adenocarcinoma	2 (4.44%)	T4: 100% (*n* = 2)	-	-	60.5 ± 38.9	2:0	R0	10.5 ± 2.1	0.55‰
MiNEN	1 (2.22%)	in situ and T3	-	-	63	0:1	R0	79	0.27‰

Abbreviations: AMN—appendiceal mucinous neoplasm; MiNEN—mixed neuroendocrine-non-neuroendocrine neoplasm; NET—neuroendocrine tumour. * Prevalence calculated for 1000 appendectomies. ** A single case was lacking data, due to the condition of the surgical specimen.

**Table 4:** Presence of CRN among cases diagnosed with mucinous and malignant appendiceal neoplasm.

Association of CRNs with Mucinous and Malignant Appendiceal Neoplasms				
Histological Subtype	Number of Cases	Presence of CRN	Benign	Malignant
AMN	20	35% (*n* = 7)	71.4% (*n* = 5)	28.6% (*n* = 2)
NEN	18	44.4% (*n* = 8)	25% (*n* = 2)	75% (*n* = 6)
Adenocarcinoma	4	75% (*n* = 3)	0% (*n* = 0)	100% (*n* = 3)
Goblet cell adenocarcinoma	3	33.3% (*n* = 1)	100% (*n* = 1)	0% (*n* = 0)

Abbreviations: AMN—appendix mucinous neoplasm; CRN—colorectal neoplasm; NEN—neuroendocrine neoplasm.

**Table 5:** Clinicopathological characteristics of the cases included in the study.

Features	Benign (*n* = 26)		Mucinous and Malignant (*n* = 45)			*p*-Value
	Number of Cases	Ratio (%)	Number of Cases		Ratio (%)	
**Sex**						*0.27*
Male	14	53.85%	17		37.78%	
Female	12	46.15%	28		62.22%	
**Grade (G)**			NA *	1	2.22%	*0.76*
NA	19	73.08%	G1	39	86.67%	
Low-grade	7	26.92%	G2	4	8.89%	
High-grade	0	0%	G3	1	2.22%	
**T-stage**		-				***<0.001***
NA			1		2.22%	
Tis	NA		12		26.67%	
T1	NA		12		26.67%	
T2	NA		1		2.22%	
T3	NA		7		15.56%	
T4	NA		13		28.89%	
**M-stage**						
M0	NA	-	42		93.33%	
M1	NA		3		6.67%	
**Resection**						
R0	26	100%	39		86.67%	
R1	0	0%	6		13.33%	*0.101*
**Spread**						
Vascular	NA	-	2		4.44%	***0.017***
**Presence of CRC**	12	46.15%	11		24.44%	***0.031***
Localisation of CRC **						***0.003***
Right colon	9	34.62%	6		13.33%	
Left colon	5	19.23%	3		6.67%	
Unknown	-	-	2		4.44%	
	Mean; median	Range	Mean; median		Range	
**Age (years)**	63.9; 69.5	30–90	53.6; 55		16–88	***0.046***
**OS (months)**	25.2; 14	0–101	38.1; 34		0–108	*0.401*

Abbreviations: CRC—colorectal carcinoma; G—grade; NA—not applicable; OS—overall survival. * A single case was lacking data, due to the condition of the surgical specimen. ** CRC was present in 12 benign appendiceal neoplasm cases; however, in 2 cases, there have been 2 separate CRC diagnoses both in the right and left colon, resulting in 2 additional CRC localisations. Bold letters are to indicate significant results.

## Data Availability

The data presented in this study are available on request from the corresponding author.

## Abbreviations

The following abbreviations are used in this manuscript:

AMN	Appendiceal mucinous neoplasm
CRC	Colorectal carcinoma
CRN	Colorectal neoplasm
CT	Computed tomography
HAMN	High-grade appendiceal mucinous neoplasm
HE	Hematoxylin and eosin
HP	Hyperplastic polyp
ICD-10	International Classification of Diseases, Tenth Revision
LAMN	Low-grade appendiceal mucinous neoplasm
MiNEN	Mixed neuroendocrine-non-neuroendocrine neoplasm
NA	Not applicable
NEN	Neuroendocrine neoplasm
NET	Neuroendocrine tumour
OS	Overall survival
SD	Standard deviation
SSL	Sessile serrated lesion
TNM	Tumour, Node, Metastasis
TSA	Traditional serrated adenoma
TVA	Tubulovillous adenoma
WHO	World Health Organization

## References

[1] Constantin M., Petrescu L., Mătanie C., Vrancianu C.O., Niculescu A.G., Andronic O., Bolocan A. (2023). The vermiform appendix and its pathologies. Cancers.

[2] Köhler F., Matthes N., Rosenfeldt M., Kunzmann V., Germer C.T., Wiegering A. (2023). Neoplasms of the appendix. Dtsch. Arztebl. Int..

[3] Kamalı G., Ulusoy C., Nikolovski A., Eğin S., Kamalı S. (2022). Uncommon causes of acute appendicitis: Retrospective analysis of 6785 histopathological findings in a tertiary center. Turk. J. Trauma Emerg. Surg..

[4] Singh H., Koomson A.S., Decker K.M., Park J., Demers A.A. (2020). Continued increasing incidence of malignant appendiceal tumors in Canada and the United States: A population-based study. Cancer.

[5] Orchard P., Preece R., Thomas M.G., Dixon S.W., Wong N.A.C.S., Chambers A.C., Messenger D.E. (2022). Demographic trends in the incidence of malignant appendiceal tumours in England between 1995 and 2016: Population-based analysis. BJS Open.

[6] Osueni A., Chowdhury Y.S. Appendix Cancer. StatPearls [Internet]. https://www.ncbi.nlm.nih.gov/books/NBK555943/.

[7] da Silva Abreu R.P.N. (2018). Appendiceal neuroendocrine tumors: Approach and treatment. J. Coloproctol..

[8] Elkbuli A., Sanchez C., McKenney M., Boneva D. (2019). Incidental neuro-endocrine tumor of the appendix: Case report and literature review. Ann. Med. Surg..

[9] WHO Classification of Tumours Editorial Board (2019). Digestive System Tumours: WHO Classification of Tumours.

[10] de Moortele M.V., De Hertogh G., Sagaert X., Van Cutsem E. (2020). Appendiceal cancer: A review of the literature. Acta Gastroenterol. Belg..

[11] Benedix F., Reimer A., Gastinger I., Mroczkowski P., Lippert H., Kube R. (2010). Primary appendiceal carcinoma-epidemiology, surgery and survival: Results of a German multi-center study. Eur. J. Surg. Oncol..

[12] Lin W.T., Wang Y.H., Chen W.Y., Lao W.T. (2022). Appendiceal mucinous tumor presenting as recurrent bowel obstruction. Diagnostics.

[13] González Bayón L., Martín Román L., Lominchar P.L. (2023). Appendiceal mucinous neoplasms: From clinic to pathology and prognosis. Cancers.

[14] Kangaspunta H., Tahkola K., Wirta E.V., Kotaluoto S., Laukkarinen J., Ukkonen M. (2020). Preoperative computed tomography is poor in detecting tumors of the appendix among patients with acute appendicitis: A cohort study of 5224 appendectomies. J. Trauma Acute Care Surg..

[15] Chawrylak K., Leśniewska M., Mielniczek K., Sędłak K., Pelc Z., Kobiałka S., Pawlik T.M., Polkowski W.P., Rawicz-Pruszyńsk K. (2024). Current status of treatment among patients with appendiceal tumors—Old challenges and new solutions?. Cancers.

[16] Carr N.J., Bibeau F., Bradley R.F., Dartigues P., Feakins R.M., Geisinger K.R., Gui X., Isaac S., Milione M., Misdraji J. (2017). The histopathological classification, diagnosis and differential diagnosis of mucinous appendiceal neoplasms, appendiceal adenocarcinomas and pseudomyxoma peritonei. Histopathology.

[17] Mikaeel R.R., Young J.P., Tapia Rico G., Hewett P.J., Hardingham J.E., Uylaki W., Horsnell M., Price T.J. (2021). Immunohistochemistry features and molecular pathology of appendiceal neoplasms. Crit. Rev. Clin. Lab. Sci..

[18] Connor S.J., Hanna G.B., Frizelle F.A. (1998). Appendiceal tumors: Retrospective clinicopathologic analysis of appendiceal tumors from 7970 appendectomies. Dis. Colon. Rectum.

[19] Rossi A., Patel M.N. (2023). Appendiceal neoplasms—A practical guide. J. Surg. Oncol..

[20] Leonards L.M., Pahwa A., Patel M.K., Petersen J., Nguyen M.J., Jude C.M. (2017). Neoplasms of the appendix: Pictorial review with clinical and pathologic correlation. Radiographics.

[21] Bahmad H.F., Aljamal A.A., Alvarez Moreno J.C., Salami A., Bao P., Alghamdi S., Poppiti R.J. (2021). Rising incidence of appendiceal neoplasms over time: Does pathological handling of appendectomy specimens play a role?. Ann. Diagn. Pathol..

[22] Kunduz E., Bektasoglu H.K., Unver N., Aydogan C., Timocin G., Destek S. (2018). Analysis of appendiceal neoplasms on 3544 appendectomy specimens for acute appendicitis: Retrospective cohort study of a single institution. Med. Sci. Monit..

[23] Núñez-Rocha R.E., Girón F., Rodríguez L., Camargo-Gómez D., Restrepo-Bonilla C., Panqueva R.D.P.L., Cadena M., Nassar R., Herrera-Almario G.E., Hernández-Restrepo J.D. (2023). Incidence of appendiceal neoplasms in appendectomy patients. BMC Surg..

[24] Ozdemir H., Ozdemir Z.U., Gul M.O. (2023). Incidental appendiceal neoplasms: Single-centre results. Indian J. Cancer.

[25] Süleyman M., Senlikci A., Durhan A., Kosmaz K. (2023). Incidental presentation of appendix neuroendocrine tumor: Long-term results from a single institution. Ulus. Travma Acil Cerrahi Derg..

[26] Chezar K., Minoo P. (2022). Appendiceal sessile serrated lesions are distinct from their right-sided colonic counterparts and may be precursors for appendiceal mucinous neoplasms. Hum. Pathol..

[27] Zhang H.W., Jiang Y., Huang Z.Y., Zhou X.C. (2023). Analysis of surgical treatment of appendix neuroendocrine neoplasms-17 years of single-center experience. World J. Surg. Oncol..

[28] Rencuzogullari A., Atar C., Topal U., Coğal İ., Saritas A.G., Yalav O., Dalci K., Eray İ.C. (2023). Analysis of appendiceal neoplasms in 1423 appendectomy specimens: A 10-year retrospective cohort study from a single institution. Rev. Assoc. Med. Bras.

[29] Sahin N., Ozyalvac F.T., Donmez T., Surek A., Sahin E.A., Calis G., Bulut S., Aydin H., Kabuli H.A., Gumusoglu A.Y. (2024). The incidence of incidental neoplasia in pathology samples of patient who underwent appendectomy due to acute appendicitis. A single center experience: 6446 cases. Iran. J. Med. Sci..

[30] Hara K., Saito T., Hayashi T., Yimit A., Takahashi M., Mitani K., Takahashi M., Yao T. (2015). A mutation spectrum that includes GNAS, KRAS and TP53 may be shared by mucinous neoplasms of the appendix. Pathol. Res. Pract..

